# Co-Benefits Analysis of Buildings Based on Different Renewal Strategies: The Emergy-Lca Approach

**DOI:** 10.3390/ijerph18020592

**Published:** 2021-01-12

**Authors:** Wenjing Cui, Jingke Hong, Guiwen Liu, Kaijian Li, Yuanyuan Huang, Lin Zhang

**Affiliations:** 1 School of Management Science and Real Estate, Chongqing University, Chongqing 400044, China; 20170301004@cqu.edu.cn (W.C.); hongjingke@cqu.edu.cn (J.H.); likaijian@cqu.edu.cn (K.L.); 20170301015@cqu.edu.cn (Y.H.); 2 School of Management Engineering, Shandong Jianzhu University, Jinan 250101, China; zhanglin2007@sdjzu.edu.cn

**Keywords:** urban renewal, co-benefits, emergy analysis, life cycle assessment (lca), refurbishment strategy, rebuilding strategy

## Abstract

Many cities have encountered challenges associated with rapid urban development, population growth and aging, in which urban renewal has become a promising option. Different renewal strategies, such as redevelopment, refurbishment and conservation, not only contributes to quality improvement and energy consumption reduction of dilapidated urban area, but also to greenhouse gas (GHG) emissions mitigation. Such integrated benefits are often termed as co-benefits. However, choosing the most co-benefits strategy to adopt requires a holistic understanding of social-economic and environmental aspects, which has been less reported in the existing literature. Under such circumstance, this article aims to shed light on the co-benefits of different renewal strategies by adopting the Emergy-Life cycle assessment method. Then, the method is applied to one case study of the refurbishment of an educational building located in Chongqing, China. Resource allocation, CO_2_ emissions and emergy-based indicators are calculated to assess the co-benefits during a 60-year research period, to compare the impacts of the complete demolition followed by a new one (rebuilding strategy) and the refurbishing of the existing building (refurbishment strategy). The case study shows that the annual emergy in the O&M phase of rebuilding strategy and refurbishment strategy were lower than existing building. Rebuilding and refurbishment strategies released approximately 59.1% and 80.6%, respectively, of the total CO_2_ emissions that would be produced by the existing building. The results reveal that substantial environmental benefits can be obtained in both the refurbishment and rebuilding strategies. On the other hand, it can be concluded that the emergy yield ratio (EYR) for the rebuilding strategy is higher than refurbishment strategy, which demonstrate the better performance of refurbishment considering that less resources are required to generate greater benefits. In addition, the value of environmental loading ratio (ELR) and emergy sustainability index (ESI) also suggests that the refurbishment strategy performs better from the perspective of the environment. Thereby, the refurbishment strategy is more suitable than the rebuilding strategy. Findings from this study can be useful to urban planners and decision-makers in choosing the most suitable strategy to improve the quality of existing buildings.

## 1. Introduction

Most cities worldwide are exposed to social-economic and environmental challenges caused by rapid industrialization, population growth and urbanization [1]. These challenges include urban dilapidation, economic decline, unreasonable utilization of land and environmental pollution [2,3,4]. Urban renewal, as a process to achieve the sustainable development in terms of economy, society and environment [5,6,7], is an ideal approach to resolve urban issues.

Urban renewal, also known as urban regeneration, offers a chance to improve the physical, social, economic and ecological conditions of decaying urban areas by three strategies: rebuilding, refurbishment and conservation [7]. One of the main strategies of urban renewal is rebuilding, which involves demolishing dilapidated buildings and replacing them with brand-new buildings, as was done in New York, USA, from 1949–1972 [8]. The rebuilding strategy enables eradication of substandard buildings and improves the land use [9,10]. Moreover, it can easily incorporate open spaces and community facilities, which gives neighborhoods in the redevelopment sites a positive externality [10,11] and reduces energy consumption [12]. However, rebuilding destroys the social fabric of cities [13] and generates a large amount of construction waste [14].

Refurbishment refers to the rehabilitation of outdated buildings through a series of efforts, to improve quality standards and function [9]. Generally, the main volume of the building was preserved in refurbishment strategy. The refurbishment strategy offers a quicker and less socially disturbing option to improve the building stock quality [10,11]. In addition, the refurbishment strategy enables building energy performance improvement through the integration of energy efficiency and renewable energy measures [15]. However, according to [16], the cost of refurbishment is similar to that of rebuilding.

The third strategy is conservation, also known as preservation or restoration, which is usually performed in heritage buildings [17]. Conservation includes a series of processes to maintain a building and preserve and protect its historical and cultural values [13]. However, most of the old building envelopes have a poor thermal insulation performance [18,19], which requires frequent repair and rehabilitation measures [17].

**Figure 1:**
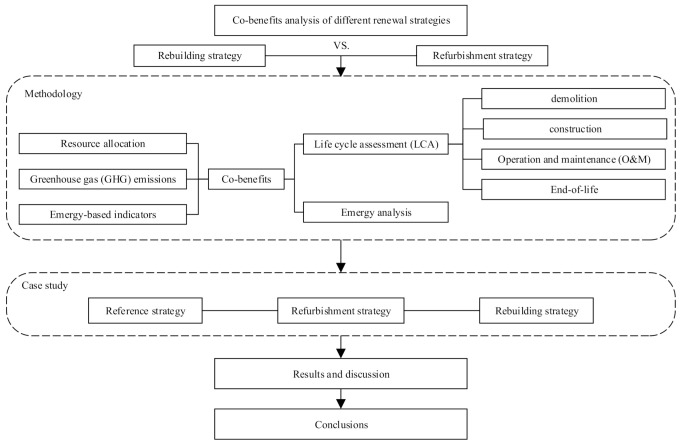
Research framework.

Although the co-benefits captured in the above three urban renewal strategies is significant, the decision which strategy to adopt is complex and requires the integration of social, economic, and environmental parameters [17]. The process of choosing the appropriate urban renewal strategy has been debated for over a century [20]. However, it remains unclear whether rebuilding, refurbishing or conserving existing buildings is the most suitable option [21]. In this context, the aims of this study are (i) to investigate the co-benefits of different renewal strategies by employing EM-LCA method (combination of emergy and LCA method); (ii) to provide a comparison between refurbishment strategy and rebuilding strategy. Figure 1 illustrates the stages of the research. After the introduction section, the review of the relevant work on the topic is discussed in Section 2. Additionally, the EM-LCA method adopted in this study is presented in Section 3. Then, a detailed case description and three strategies is provided in Section 4. Section 5 and Section 6 first focus on the results and discussion of the emergy of the building system and the greenhouse gas (GHG) emissions in every strategy, and the co-benefits of different renewal strategies are then revealed and compared. Finally, certain conclusions are drawn.

## 2. Review of Relevant Works

Several recent studies on the comparison between refurbishment strategy and rebuilding strategy were conducted around the world (Table 1). However, the results of this research were polarized. Some researchers argued that refurbishment strategy was better than rebuilding strategy in terms of cost, time, energy performance, CO_2_ emissions and other environmental indicators. A recent study on energy-saving measures was conducted by Gaspar and Santos, the results revealed that the rebuilding strategy consumed more embodied energy than the refurbishment strategy [16]. Weiler et al. calculated the embodied energy, the embodied greenhouse gases (GHGs), the energy required and the GHGs emitted during the life cycle of an individual building [22]. Accordingly, Weiler suggested that refurbishing is better than rebuilding. Hasik et al. conducted a comparative between refurbishment and new construction by a whole-building life cycle assessment, which showed that 53–75% reductions when the refurbishment was compared to rebuilding strategy in terms of acidification potential, eutrophication potential, global warming potential, ozone depletion potential, smog formation potential and non-renewable energy demand [23]. For materials and waste, the environmental impact of refurbishment strategy is better rebuilding strategy [24].

In contrast, some previous comparison studies of refurbishment and rebuilding have other different opinions. A life cycle performance for 4 refurbishment scenarios and 2 reconstruction scenarios were investigated, the results showed that the rebuilding scenarios appear to be the better choice compared to refurbishment when the total life cycle emissions intensity is considered [25]. The same results were also gained by Rønning et al., which investigated a Norwegian bank through a hybrid LCA approach [26]. In addition, for the cost, Ferreira, Pinheiro and Brito suggested the refurbishment strategy was less competitive than rebuilding strategy [27].

Based on the aforementioned review, the results of previous studies suggested that urban renewal have some co-benefits over existing buildings, such as the improvement of building quality and function, the reduction of energy consumption and the improvement of environment. However, several research gaps are identified from the current studies. The results of previous researches about whether rebuilding or refurbishing existing buildings is the better option remains unclear [21]. As Table 1 shows, these studies mainly focused on the residential building in European countries, few studies have been carried out to investigated the office building. Energy and cost were adopted in most studies as the mainly criteria to make comparison, which neglect other aspects. Moreover, numerous studies adopted the life cycle assessment (LCA) method to assess the energy consumption of different renewal strategy. LCA method only studies the environmental impacts of products or processes but neglects the impacts of other aspects, such as economic effects [28]. The decision which strategy to adopt is complex and requires a comprehensive tool to integrate social, economic, environmental, and political-institutional parameters [17].

Emergy (Em) is an environmental policy tool for evaluating the quality of resources based on the dynamics of complex systems [33]. Odum defined emergy as the total amount of one kind of available energy (exergy) that is directly or indirectly used in transformations to generate a given product or support a given service [34]. Em assesses the system performance by quantifying the emergy value of different kinds of resources, such as renewable and nonrenewable resources, labor/services, gas emissions, and liquid and solid wastes [35]. Accordingly, with Em, it is possible to develop a link between economic and ecological systems [36,37,38] and allows the direct comparison of different products and services [39,40]. In addition, the Em method can not only investigate the social-economic and environmental impacts of a system [41] but can also analyze the resources consumed to support labor and services, which is often not considered in the process of LCA [42]. The combination of Emergy and LCA (EM-LCA) is a more comprehensive sustainability assessment tool for complex systems [28,43].

Measures to mitigate the climate change and environmental pollution can bring some unintentionally positive benefits, as called co-benefits [44]. A growing number of studies having discussed the co-benefits of climate change policy at different countries, particularly China, India and Bangladesh [45,46,47,48]. The co-benefits of air quality management plan and GHG emissions reduction strategies in the Seoul metropolitan area and New Zealand were evaluated by some models [49,50]. Dong et al. studied the CO_2_ emissions and air pollutants emissions in China, and the reduction cost and co-benefits effect [51]. The building environment interventions will yield the co-benefits of mitigating climate change and promoting public health [52]. Certified green building substantially generate the co-benefits to public health, which were estimated in the six countries (the United States, China, India, Brazil, Germany and Turkey) [53]. Co-benefits to public health, such as improving the interface between humans and wildlife, reducing the risk of waterborne disease, flood-related morbidity and mortality, and psychological harm, were evaluated [54]. Furthermore, the GHG emissions co-benefits associated with water, waste and transportation usage in LEED building were also investigated in California [55]. As for the existing building, co-benefits of the residents, housing association and society in general, GHG emissions and energy consumption can be achieved after implementing retrofit technological measures [56,57,58]. The co-benefits of residential buildings in terms of the energy savings, costs and other additional benefits of renovation scenarios were investigated to support decision making [59,60]. However, few studies have been conducted relating the co-benefits of different renewal strategies with the whole life cycle approach.

## 3. Method

### 3.1. EM-LCA Approach

**Figure 2:**
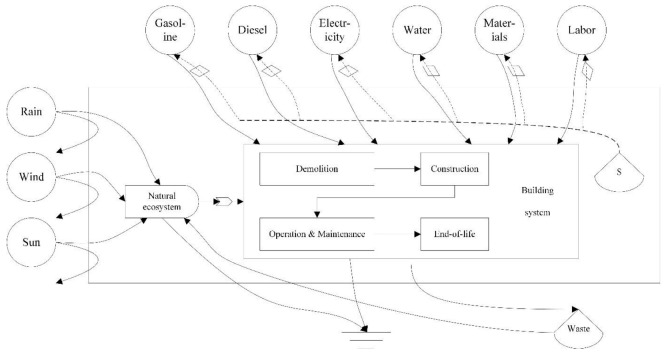
Emergy diagram of the material and energy flows.

In this study, the EM-LCA approach was applied to quantify the co-benefits of the different renewal strategies. This approach aims to offer a same quantitative framework among different resources, energy and human services [36], which is a more comprehensive technique than existing LCA tools [28]. Figure 2 presents an overview of the boundaries of building system and depicts the constituents, resource flows, exchange pathways, and downstream outflows. The system boundary in this research includes both spatial and life cycle process boundaries [61,62]. The spatial boundary is the three-dimensional space of a building, which includes the foundation at the bottom, the highest point and the façade of the building. The lifecycle process boundary includes all the upstream and downstream processes to establish and maintain the functions of a building [63], which contain all processes from the cradle to the grave. The lifecycle process boundary in this study extends from the demolition phase to the end-of-life phase based on the renewal of existing buildings, namely, the demolition phase, construction phase (including the material production phase, transportation phase and on-site phase), O&M phase and end-of-life phase [63]. This paper does not consider the construction/demolition waste due to the lack of data. The building system is regarded as a thermodynamic engine in which natural, social and economic resources are invested to generate products and maintain the base performance, thereby releasing pollutants to the atmosphere, water bodies and land. Accordingly, the driving energy, materials and interactions, as well as outflows and feedback of the system, are simulated as energy pathways [28].

### 3.2. Resource Allocation

Seven different types of resources have been identified in the building system input, as shown in the emergy flow diagram in Figure 2. These resources include solar irradiation, materials, electricity, water, diesel fuel, gasoline and human labor.

(1) Solar irradiation—the input of solar irradiation (Equation (1)) to urban renewal is regarded as a kind of free renewable resource invested in the building system, which can impact the indoor thermal environment of buildings [40,64].

(1) $$E_{s,c}=S\times I\times \left(1-a\right)\times t_{c}\times T_{s}$$

where $E_{s,c}$ is the solar emergy of the solar irradiation in the construction phase; S is the construction site surface (footprint); I is the annual amount of solar radiation, equal to 3.5 × 10^9^$\;J/m^{2}$ [65]; a is the surface albedo, equal to 0.7 in this study; $t_{c}$ is the construction time; and $T_{s}$ is the transformity of solar energy.

(2) Materials—the building materials are the resources invested in the system to construct a building, which mainly occurs in the construction phase [34,64]. The emergy of the system inflows can be calculated as follows (Equation (2)):

(2) $$E_{m}=\overset{n}{\underset{i=1}{\sum }}M_{i}\times T_{mi}$$

where $E_{m}$ is the solar emergy of the building materials; $M_{i}$ is the quantity of material i; and $T_{mi}$ is the transformity of material i.

(3) Electricity—the emergy flow of the electricity (Equation (3)) consumed during the life cycle is calculated as:

(3) $$E_{e}=W\times T_{e}$$

where $E_{e}$ is the solar emergy of the electricity required in the building construction, O&M and end-of-life phases; W is the quantity of electricity obtained from the results of energy consumption simulation with EnergyPlus 8.7(developed by Department of Energy and Lawrence Berkeley National Laboratory, Berkeley, California, US); and $T_{e}$ is the transformity of electricity.

(4) Water—the emergy flow of the water (Equations (4) and (5)) used during the building lifetime can be calculated as:

(4) $$E_{w,c}=V\times \rho_{w}\times G\times T_{w}\;$$

where $E_{w,c}$ is the solar emergy of the water consumed in the building demolition, construction and end-of-life phases; V is the required water volume; $\rho_{w}$ is the water density; G is the Gibbs free energy of water, which equals 4.92 J/g [66]; and $T_{w}$ is the transformity of water.

(5) $$E_{w,o}=V_{a}\times N_{n}\times t_{o}\times \rho_{w}\times G\times T_{w}$$

where $E_{w,o}$ is the solar emergy of the water consumed in the building O&M phase; $V_{a}$ is the required water volume of one person per day, which equals 20 L/d/p in this study [40]; $N_{n}$ is the number of employees, which equals 200 in this study; and $t_{o}$ is the total number of working days per year, which is assumed to be 300 days in this study.

(5) Diesel fuel—the consumption of diesel fuel mostly occurs during the demolition, construction and end-of-life phases, the emergy of diesel fuel (Equation (6)) can be calculated as:

(6) $$E_{d}=M_{d}\times c_{d}\times T_{d}\;$$

where $E_{d}$ is the solar emergy of the diesel fuel consumed; $M_{d}$ is the quantity of diesel fuel consumed in the building life cycle; $c_{d}$ is the calorific value of diesel fuel; and $T_{d}$ is the transformity of diesel fuel.

(6) Gasoline—the solar emergy of gasoline (Equation (7)) is calculated as:

(7) $$E_{g}=M_{g}\times c_{g}\times T_{g}\;\;$$

where $E_{g}$ is the solar emergy of gasoline; $M_{g}$ is the quantity of gasoline consumed in the building life cycle; $c_{g}$ is the calorific value of gasoline; and $T_{g}$ is the transformity of gasoline.

(7) Human labor—the emergy of human labor (Equation (8)) is mainly required during the building demolition, construction and end-of-life phases. The method for calculating the emergy of labor can be represented as follows [64]:

(8) $$E_{l}=N_{h}\times N_{n}\times t_{t}\times T_{l}$$

where $E_{l}$ is the emergy equivalent of human labor; $N_{h}$ is the number of working hours per day of one employee, which is 8 h in this study; $N_{n}$ is the number of employees, which equals 10 in this study; $t_{t}$ are the working days required in the life cycle process; and $T_{l}$ is the transformity of labor.

### 3.3. GHG Emissions

Compared with the material transportation and on-site phases, the CO_2_ emissions in the raw material manufacturing phase account for 80–90% of the total emissions [67]. Therefore, the CO_2_ emissions in the construction phase only include those in the raw material manufacturing phase. The CO_2_ emissions in the O&M phase mainly refer to the emissions generated by electricity under the case conditions (Equation (9)), which can be calculated as:

(9) $$G_{CO_{2}}=\overset{n}{\underset{i=1}{\sum }}M_{i}\times E_{CO_{2}-eq,\;i}\;\;$$

where $G_{CO_{2}}$ is the amount of CO_2_ emissions; $M_{i}$ is the quantity of material i; and $E_{CO_{2}-eq}$ is the emission factor of the different building materials i. Table 2 lists the emission factors of the different building materials.

### 3.4. The Emergy-Based INDICATORS

The resources input to the building system can be divided into three aspects depending on the source type, i.e., renewable, nonrenewable and purchased resources [72]. The emergy-based indicators to assess the building system performance in this paper are the emergy yield ratio (EYR), environmental loading ratio (ELR) and emergy sustainability index (ESI), as summarized in Table 3.

## 4. Case Study

A six-story educational building located in Chongqing, China, was adopted as a case study. The building was constructed in 1994 and had been in use for approximately 25 years. The building was chosen as the case study because it was deemed suitable to be renovated due to its obsolete and poor performance. The building has a gross floor area of 6500 m^2^, including a terrace on the third floor. The building story height is 3.6 m, and the structure consists of bricks and reinforced concrete elements such as foundation footings, columns, beams, slabs and staircases. Its walls are constructed of red clay bricks, laid with cement mortar, which have been rendered and painted. The windows are single-glazing windows, and the roof is neither insulated nor waterproof. Inside the building, the floors are finished with ceramic tiles and terrazzo concrete. The interior walls and ceilings are plastered and painted.

### 4.1. Reference Strategy

Conservation strategies are often applied to historical buildings with a historical or cultural value. Therefore, a comparison was conducted of the refurbishment and rebuilding strategies.

**Figure 3:**
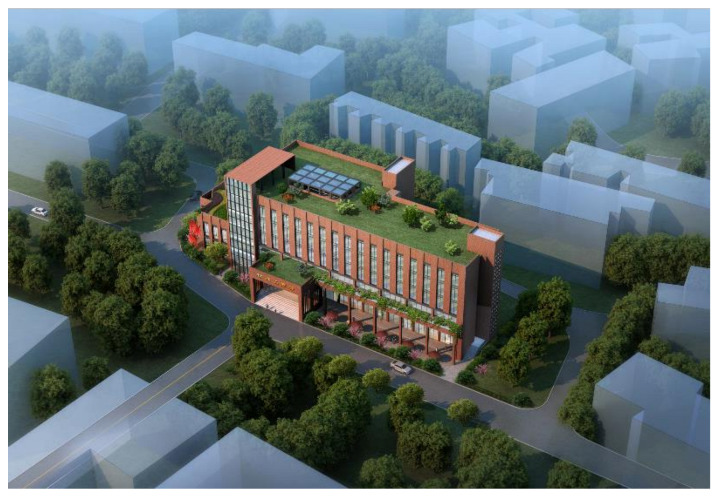
Aerial view of the building.

The reference strategy is existing building before refurbishment or rebuilding. The list of required construction materials and technical specifications was obtained from project documents as developed by the architect and engineers. Based on the physical and functional parameters of the building, this paper assumed that the remaining life of the existing building is 10 years. Figure 3 shows an aerial view of the building.

### 4.2. Refurbishment Strategy

**Figure 4:**
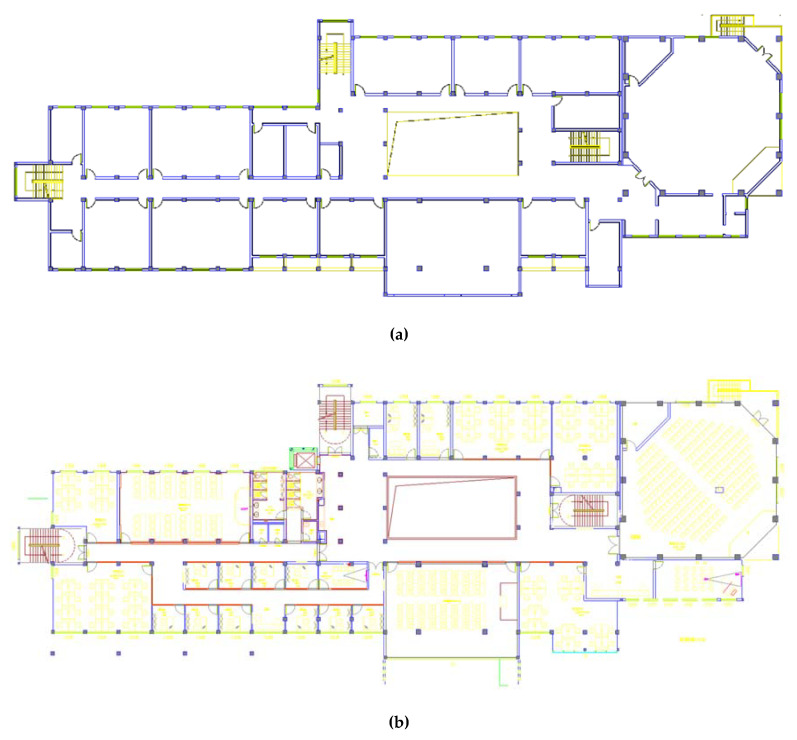
Second-floor plan. (**a**) Second-floor plan before refurbishment. (**b**) Second-floor plan after refurbishment.

Refurbishment strategy included improving the building structure, functions and energy performance such as the addition of insulation to the walls, floors, and roof, the installation of new windows, and the fitting of energy-efficient electric appliances and lighting systems. The original building structure and foundation were preserved, but minor structural repairs were considered to strengthen and extend the building life span. The insulation performance of the building envelope may deteriorate over time due to several factors, such as the type of insulation, workmanship and level of exposure to weather conditions [73]. In this project, additional insulation to the walls, floors, and roof was suggested. It was proposed that all existing windows should be replaced due to their poor performance in thermal transmittance. Electric appliances such as air conditioners and lighting systems with high energy efficiency levels and savings were recommended. The life span of the building after refurbishment was assumed to be 20 years [74]. Figure 4 shows the plan of the second floor before and after refurbishment.

### 4.3. Rebuilding Strategy

Rebuilding strategy in this case refers to the complete demolition of the existing building, thereby constructing a new building. According to national building regulations and standards, the new building must satisfy specific energy savings and seismic requirements. The new building would use reinforced concrete (RC) elements for its foundation, columns, beams and slabs. The interior layout, electric appliances and lighting systems were designed similar to those in the refurbishment strategy. The other substructures of the new building remained similar to those of the existing building for the sake of simplification. The lifespan of a new office building is often quoted to range from 40–75 years [75]. Therefore, the lifespan in this study was assumed as 60 years [26,28].

### 4.4. Data Collection

The renewal strategy data were obtained from relevant construction documents and project information. The collection of building data for the rebuilding strategy is time consuming and difficult due to the lack of construction documents. Therefore, this study obtained data from the documents pertaining to the existing building and the refurbishment strategy. In other words, this paper combines the documents of the existing building and information of the refurbishment strategy to obtain the construction documents for a new building in order to better calculate the co-benefits.

Once the list of building material quantities was compiled with the material flows in each strategy, the emergy of the input and output materials was calculated. The CO_2_ emissions in the different renewal measures was obtained by field measurements, simulation software from EnergyPlus 8.7 and the literature. Data on the solar transformity and certain emergy calculation processes were acquired from the literature. To conduct a better comparison, this study adopted 60 years as the research period, during which each strategy was implemented.

## 5. Results

The major emergy flows and co-benefits of the two renewal strategies are individually addressed and examined to compare their impacts and suggest the most suitable strategy.

### 5.1. Emergy Flow

#### 5.1.1. Resource Allocation

An overview of the weight and emergy of resources required in the rebuilding strategy or the refurbishment strategy is presented on Table 4 and Table 5. It is clear that comparing the lower emergy flow during demolition and end-of-life phase, 32.74% and 14.63% of the emergy flow was caused during the construction phase, 67.25% and 85.35% during the O&M phase for the new building and the building after refurbishment, respectively. The emergy of the resources during the construction phase mainly consists of cement, concrete and gravel. The emergy of water and electricity during the O&M phase is higher than other resources, which agrees with the function of the building as an educational building. The results suggest that the consumption of resource during the O&M phase is the high and the importance of the resource management in O&M phase [64].

**Figure 5:**
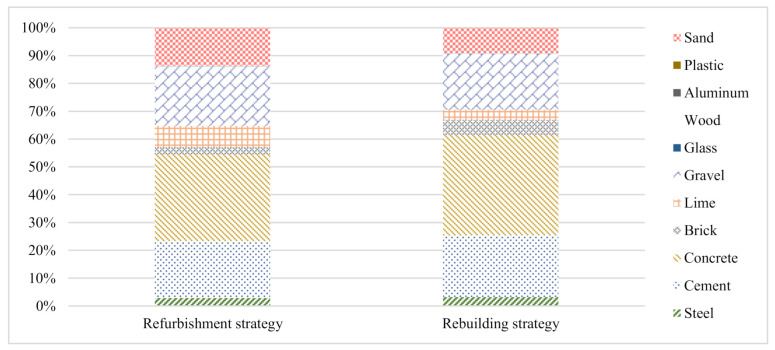
The weight of the building materials in the construction phase.

Further, analysis of the weight of building materials in the construction phase reveals the quantities of materials such as concrete, cement, brick, lime, and sand used in the construction phase, which account for more than 80% of the total weight (Figure 5). The weight of concrete in the refurbishment strategy accounts for approximately 31.20% of the total weight, while that in the rebuilding strategy accounts for approximately 36.01%, which implies that the concrete consumption level is the highest [76]. The relatively less consumed materials include wood, aluminum, and plastics. These results are consistent with the general understanding in the construction industry that the consumption of sand, concrete cement and bricks is high in the construction phase Gaspar and Santos [16].

#### 5.1.2. GHG Emission

To provide a complete picture of the environmental performance of the different renewal strategies, the CO_2_ emissions of the resources were calculated. Table 6 and Table 7 contain detailed information on the CO_2_ emissions for the rebuilding and refurbishment strategies. It is clear that 11.5% of the CO_2_ emissions were generated in the building construction phase of the rebuilding strategy and 12.1% were generated in the refurbishment strategy. In the O&M phase, the CO_2_ emissions were 88.5% in the rebuilding strategy and 87.9% in the refurbishment strategy. Notably, O&M phase is the largest contributor to CO_2_ emissions by comparing the life cycle CO_2_ emissions, followed by the construction phase [62,63,77]. The CO_2_ emissions of steel, concrete and cement together accounted for above 70% of the total emissions in both strategies. Relatively low emissions are associated with building materials such as wood, aluminum, glass, etc. In the O&M phase, electricity is the most important factor impacting the environment due to the character of the consumed resources. The results indicate that in the construction phase, the type and quantity of building materials used has a far-reaching impact on the total carbon dioxide emissions [78].

### 5.2. Co-Benefits

#### 5.2.1. Resource Allocation

**Figure 6:**
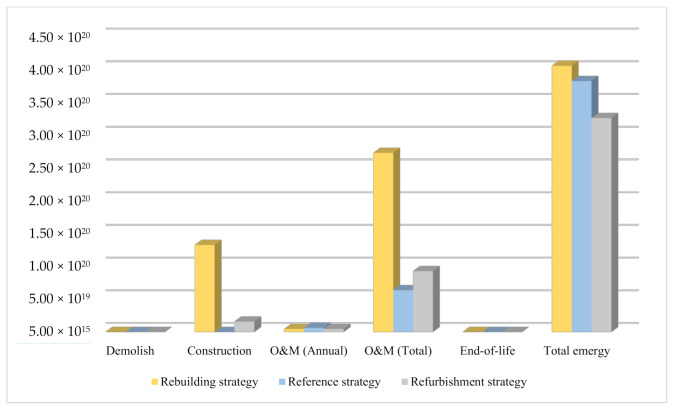
Emergy values in the life cycle phases of the different strategies.

The emergy of the resources invested in the building system is illustrated in Figure 6. It is clear that the emergy is highest during the construction phase of the rebuilding strategy, followed by the refurbishment strategy, and finally the existing building, which indicated that the large number of materials were required in the construction phase. The annual emergy in O&M phase of rebuilding strategy, reference strategy and refurbishment strategy were 4.56 × 10^18^ seJ, 6.39 × 10^18^ seJ, 4.65 × 10^18^ seJ, respectively, which concluded that resource consumption of rebuilding strategy and refurbishment strategy in O&M phase is lower than existing building and the co-benefits of renewal strategies over existing building. Emergy of the new building during O&M phase is lower than refurbished building. However, the total emergy in the rebuilding strategy (4.07 × 10^20^ seJ) is the largest one among the three strategies as newer materials are used for the building, in line with a more complex practice and higher energy saving standard [16]. The minimum emergy value was attained for the refurbishment strategy, which shown the co-benefits of refurbishment strategy is higher than the rebuilding strategy.

#### 5.2.2. GHG Emissions

**Figure 7:**
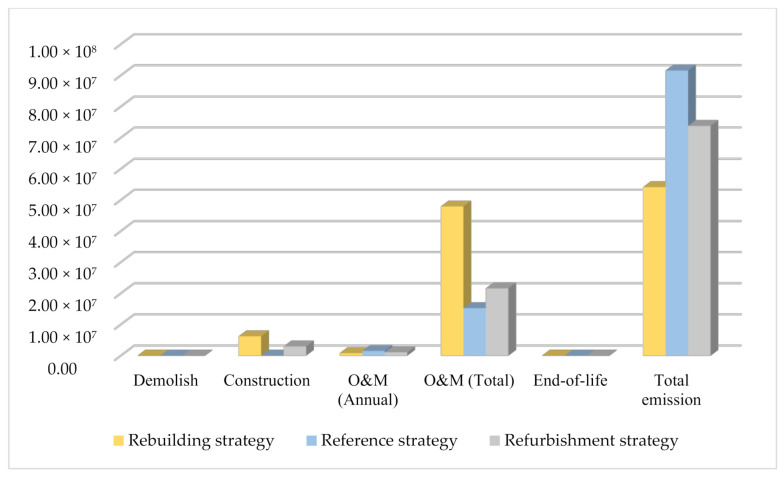
The CO_2_ emissions in the life cycle phases of the different strategies.

Figure 7 shows the CO_2_ emissions in each phase of the life cycle and the total emissions. It is clear that rebuilding strategy contribute more CO_2_ emissions than refurbishment strategy during construction phase as large amount of building materials were used. During the O&M phase, the annual CO_2_ emissions of rebuilding, refurbishment and reference strategy were estimated as 7.99 × 10^5^ kg, 1.08 × 10^6^ kg and 1.53 × 10^6^ kg, respectively. Meanwhile the total CO_2_ emissions of rebuilding strategy during O&M phase is the most, followed by refurbishment strategy due to the different O&M time. It is clear that the rebuilding and refurbishment strategies released approximately 59.1% and 80.6%, respectively, of the total CO_2_ emissions that would be produced by the existing building. Thus, the total CO_2_ emissions over the entire building life cycle is lower than existing building for both renewal strategies. The comparison of life cycle CO_2_ emissions demonstrated that substantial environmental benefits can be obtained in both the refurbishment and rebuilding strategies [60,77]. On the other hand, the total CO_2_ emissions of rebuilding strategy is lower than refurbishment strategy during the research period, which is clearly in favor of the rebuilding strategy [25,26].

#### 5.2.3. The Emergy-Based Indicators

Table 8 summarizes the total emergy invested in the building system and the CO_2_ emissions released by the system after implementing the different renewal strategies during the research period (60 years). The results indicate that the best strategy to realize the highest co-benefits is the reference strategy as consumed the least resources. On the other hand, the best strategy is the rebuilding strategy in terms of the total GHG emissions. Therefore, certain emergy-based indicators were calculated to explain the co-benefits.

Table 9 provides a detailed information of the emergy-based indicators about three strategies. By comparing the different strategies, it can be concluded that the EYR for the rebuilding strategy, reference strategy and refurbishment strategy is 1.12, 1.08 and 1.18, respectively, which indicate that the implementation of building refurbishment measures performs better considering that less resources are required to generate greater benefits. The ELR (Environmental loading ratio) and ESI (Emergy sustainability index) reflect the environmental performance of the building system. It is clear that the ELR for the rebuilding strategy is 9.51, which is higher than that for the refurbishment strategy, and the ESI for the rebuilding strategy is lower than that for the refurbishment strategy, which indicates that the refurbishment strategy performs better from the perspective of the environment. Therefore, the refurbishment strategy is more suitable than the rebuilding strategy.

## 6. Discussion

The proposed method was used to investigate the emergy flow and co-benefits of different urban renewal strategies. The values for the resource allocation, GHG emissions and the emergy-based indicators of three renewal strategies were presented. The emergy of renewal strategies showed that the refurbishment or rebuilding of existing building resulted in a reduction in the annual emergy and GHG emissions of the building. The reason was perhaps that the energy performance of envelope and HVAC system was improved after enhancing thermal performance of the external walls, roof, door and other envelope elements and replace the low energy efficient windows and HVAC system with a high one [64,79,80]. The results were also consistent with the study of Andric and Jradi, indicating that the renewal strategies were indeed better than that of existing building [64,81].

For refurbishment strategy and rebuilding strategy, it is clearly shown that the rebuilding strategy has the higher total emergy value, while the value of per year is lower than refurbishment strategy. The reason for it may be the use of large amount of building materials and machines in construction phase and the more complex practice and higher energy saving standard [16]. From the perspective of GHG emissions, the total GHG emissions of rebuilding strategy (5.41 × 10^7^ kg) is lower than refurbishment strategy (7.38 × 10^7^) during the research period, which means rebuilding strategy have a better performance than refurbishment. The results were also consistent with the research of Feng et al. and Rønning et al., which in favor of the rebuilding strategy [25,26]. However, the emegy-based indicators of rebuilding strategy perform worse than refurbishment strategy. The reason was perhaps that the total emergy of rebuilding strategy is higher than that of refurbishment, which resulted in the reduction of whole performance.

## 7. Conclusions

Urban renewal is a promising solution for coping with city aging and urban environment through different strategies, such as redevelopment, refurbishment and conservation. However, selecting the appropriate strategy for sustainable urban renewal remains unclear based on the existing literature. Under such circumstances, the EM-LCA approach combining Em and LCA was adopted in this study to compare the co-benefits of rebuilding strategy with refurbishment strategy based on a case study. Emergy flows and GHG emissions in different strategies and their co-benefits over existing building were analyzed during the life cycle of the building in the case study.

The results in this case demonstrate that a large quantity of materials was consumed in the construction phase, among which concrete, cement and brick accounted for a large portion. The annual emergy in O&M phase of rebuilding strategy and refurbishment strategy is lower than the existing building. However, the total emergy of rebuilding strategy is higher than the existing building because of the large amount of materials consumption in construction phase, which suggest that the refurbishment strategy is a better choice. In terms of GHG emissions, the highest CO_2_ emissions occurred in the O&M phase, which accounted for more than 85% of the CO_2_ emissions during the whole life cycle. The rebuilding strategy and refurbishment strategy released lower GHG emissions, which account for 59.1% and 80.6% of the total GHG emissions that produced by the existing building. On the other hand, it can be concluded that the EYR for the rebuilding strategy is higher than refurbishment strategy, which demonstrate the better performance of refurbishment considering that less resources are required to generate greater benefits. Additionally, the value of ELR and ESI also suggests that the refurbishment strategy performs better from the perspective of the environment. Thereby, the refurbishment strategy is more suitable than the rebuilding strategy.

This paper presents an effective and comprehensive method to assess the impact of different renewal strategies, which is highly relevant and useful for many future renewal projects. Furthermore, the findings of this study may be helpful to decision-makers when choosing the appropriate strategy and have a far-reaching effect on policy implementation. Additionally, this study may be useful to academics, as it possibly represents another study area, such as the comparison of the aforementioned three strategies in community level. However, the study had some important limitations as the emergy flow and GHG emissions of renewal strategies are calculated using current data. Moreover, it did not consider the social impacts, which is also significant for implementing urban renewal projects.

## Figures and Tables

**Table 1:** Recent studies on comparison of rebuilding and refurbishment.

Literature Source	Country	Building Type	Method	Measurement Criteria	Main Conclusions
La Fleur, Rohdin [29]	Sweden	Residential	Life cycle assessment (LCA)	Life cycle cost (LCC)	The cost of the new building is higher compared to energy renovation.
Gaspar and Santos [16]	Portugal	Residential	cradle-to-gate	Energy Weigh	Refurbishment was a more sustainable strategy than rebuilding.
Weiler, Harter [22]	Germany	Residential	LCA	Energy	It is better to refurbish an existing building than to demolish it and reconstruct.
Morelli, Harrestrup [30]	Denmark	Residential	Two-fold evaluation	Cost of conserved energy	Renovating the building will be an economically sensible solution as compared to demolishing and erecting a new one.
Marique and Rossi [21]	Brussels	Office	LCA	Energy	The retrofitting of the building is significantly less harmful than its complete demolition/reconstruction.
Ferreira, Duarte Pinheiro [27]	Portugal	Palace	cradle-to-gate	Energy Cost	Refurbishment was environmentally more positive than the new equivalent construction. For cost, refurbishment was less competitive than demolition followed by a new equivalent construction.
Rønning, Vold [26]	Norway	Norwegian Bank	LCA	Energy	From a climate point of view the most favourable strategy was to replace the existing construction and build a new one.
Elmezaini [31]	Gaza	Al-Amin Mosque		Time Cost	A cautious repairing program was successfully adopted which saved time and cost.
Itard and Klunder [32]	Netherlands	Residential	LCA	Material Energy Water use Demolition waste	The transformation of the existing housing stock is found to be a much more environmentally efficient way to achieve the same result than are demolition and rebuilding.
Feng, Liyanage [25]	Canada	Residential	LCA+ Building information modelling (BIM)	Energy	Renovations lead to much lower embodied emissions compared to reconstruction. When the total life cycle emissions intensity is considered, the reconstruction options also appear to be the better choice compared to renovations at the full 50-year project assessment period.

**Table 2:** Emission factors of the different materials.

No.	Main Building Materials	Unit	Reference	CO_2_ Emissions (kg/unit)
1	Steel	kg	Peng [68]	2.208
2	Cement	kg	Peng [68]	0.894
3	Concrete	kg	Peng [68]	0.242
4	Brick	kg	Peng [68]	0.200
5	Lime	kg	Peng [68]	1.200
6	Gravel	kg	Peng [68]	0.002
7	Glass	kg	Peng [68]	1.400
8	Wood	kg	Peng [68]	0.200
9	Aluminum	kg	Peng [68]	1.407
10	Sand	kg	Mao, Shen [69]	0.0069
11	Diesel	L	Zhan, Liu [70]	2.730
12	Gasoline	L	Zhan, Liu [70]	2.260
13	Electricity	kWh	National development and reform commission in China (NDRC [71]	0.9929

**Table 3:** Emergy indices and description.

Emergy Indices	Description
R	Renewable resources
N	Nonrenewable resources
F	Emergy feedback
Y	Emergy yield: N+R+F
EYR ^a^	Emergy yield ratio: Y/F
ELR	Environmental loading ratio: (N+F+EL)/R
ESI	Emergy sustainability index: EYR/ELR

^a^ Emergy indices were adapted from [33,34].

**Table 4:** Emergy table for the rebuilding strategy.

Item	Resources (unit)	Transformity (seJ/unit)	Reference	Demolition		Construction		O&M			End-of-Life		Total	Emergy (Unit)	
				Raw Data	Emergy (seJ)	Raw Data	Emergy (seJ)	Annual	Total (60 years)	Emergy (seJ)	Raw Data	Emergy (seJ)			Type
1	Steel (g)	1.40 × 10^9^	Odum [34]			5.30 × 10^8^	7.42 × 10^17^						5.30 × 10^8^	7.42 × 10^17^	F
2	Cement (g)	3.30 × 10^10^	Odum [34]			3.65 × 10^9^	1.21 × 10^20^						3.65 × 10^9^	1.21 × 10^20^	F
3	Concrete (g)	5.08 × 10^8^	Wang and Zhang [72]			5.93 × 10^9^	3.01 × 10^18^						5.93 × 10^9^	3.01 × 10^18^	F
4	Brick (g)	2.52 × 10^9^	Wang and Zhang [72]			9.03 × 10^8^	2.28 × 10^18^						9.03 × 10^8^	2.28 × 10^18^	F
5	Lime (kg)	1.28 × 10^12^	Odum [34]			6.05 × 10^5^	7.74 × 10^17^						6.05 × 10^5^	7.74 × 10^17^	F
6	Gravel (kg)	1.27 × 10^12^	Andrić, Pina [64]			3.30 × 10^6^	4.20 × 10^18^						3.30 × 10^6^	4.20 × 10^18^	N
7	Glass (g)	8.40 × 10^8^	Odum [34]			2.32 × 10^6^	1.95 × 10^18^						2.32 × 10^6^	1.95 × 10^15^	F
8	Wood (t)	4.40 × 10^4^	Odum [34]			4.06 × 10^1^	1.79 × 10^6^						4.06 × 10^1^	1.79 × 10^6^	R
9	Aluminum (kg)	1.60 × 10^10^	Odum [34]			3.98 × 10^2^	6.37 × 10^12^						3.98 × 10^2^	6.37 × 10^12^	F
10	Plastic (g)	3.80 × 10^10^	Odum [34]			1.98× 10^2^	7.52 × 10^12^						1.98 × 10^2^	7.52 × 10^12^	F
11	Sand (t)	1.69 × 10^12^	Reza, Sadiq [38]			1.50× 10^3^	2.54 × 10^15^						1.50 × 10^3^	2.54 × 10^15^	N
12	Diesel (J)	1.21 × 10^5^	Reza, Sadiq [38]	1.96 × 10^7^	2.37 × 10^12^	1.57 × 10^11^	1.90 × 10^16^	0	0	0	2.05 × 10^7^	2.48 × 10^12^	1.57 × 10^11^	1.90 × 10^16^	F
13	Gasoline (J)	6.60 × 10^4^	Odum [34]	2.27 × 10^7^	1.50 × 10^12^	4.98 × 10^10^	3.29 × 10^15^	0	0	0	1.29 × 10^7^	8.51 × 10^11^	4.98 × 10^10^	3.29 × 10^15^	F
14	Solar irradiation (J)	1.00	Odum [34]	0	0	7.56 × 10^12^	7.56 × 10^12^	2.17 × 10^13 a^	1.09 × 10^15^	1.09 × 10^15^	0	0	1.09 × 10^15^	1.09 × 10^15^	R
15	Water (J)	6.60 × 10^5^	Odum [34]	9.21 × 10^8^	6.08 × 10^14^	1.69 × 10^10^	1.12 × 10^16^	6.00 × 10^11^	3.00 × 10^13^	1.98 × 10^19^	7.21 × 10^8^	4.76 × 10^14^	3.00 × 10^13^	1.98 × 10^19^	R
16	Electricity (J)	8.00 × 10^4^	Odum [34]	2.91 × 10^9^	2.33 × 10^14^	1.79 × 10^11^	1.43 × 10^16^	3.12 × 10^12^	1.56 × 10^14^	1.25 × 10^19^	4.92 × 10^9^	3.94 × 10^14^	1.56 × 10^14^	1.25 × 10^19^	R
17	Labor (h)	1.36 × 10^13^	Andrić, Pina [64]	8.14 × 10^2^	1.11 × 10^16^	1.21 × 10^5^	1.65 × 10^18^	2.88 × 10^5^	1.44 × 10^7^	1.96 × 10^20^	7.92 × 10^2^	1.08 × 10^16^	1.45 × 10^7^	1.98 × 10^20^	F
Total				1.19 × 10^16^ (0%)		1.33 × 10^20^(32.74%)		2.74 × 10^20^ (67.25%)			1.16 × 10^16^ (0%)		1.53 × 10^15^	4.07 × 10^20^	

^a^ Annual solar irradiation for O&M phase in this paper were calculated by Andrić, Pina [64].

**Table 5:** Emergy table for the refurbishment strategy.

Item	Resources (unit)	Transformity (seJ/unit)	Demolition		Construction		O&M			End-of-Life		Total	Emergy (Unit)	Total Emergy (60 Years)
			Raw Data	Emergy (seJ)	Raw Data	Emergy (seJ)	Annual	Total (20 Years)	Emergy (seJ)	Raw Data	Emergy (seJ)			
1	Steel (g)	1.40 × 10^9^			2.04 × 10^8^	2.86 × 10^17^						2.04 × 10^8^	2.86 × 10^17^	8.57 × 10^17^
2	Cement (g)	3.30 × 10^10^			1.43 × 10^8^	4.73 × 10^18^						1.43 × 10^8^	4.73 × 10^18^	1.42 × 10^19^
3	Concrete (g)	5.08 × 10^8^			2.19 × 10^9^	1.11 × 10^18^						2.19 × 10^9^	1.11 × 10^18^	3.34 × 10^18^
4	Brick (g)	2.52 × 10^9^			1.79 × 10^8^	4.52 × 10^17^						1.79 × 10^8^	4.52 × 10^17^	1.36 × 10^18^
5	Lime (kg)	1.28 × 10^12^			5.42 × 10^5^	6.94 × 10^17^						5.42 × 10^5^	6.94 × 10^17^	2.08 × 10^18^
6	Gravel (kg)	1.27 × 10^12^			1.49 × 10^6^	1.90 × 10^18^						1.49 × 10^6^	1.90 × 10^18^	5.69 × 10^18^
7	Glass (g)	8.40 × 10^8^			4.93 × 10^5^	4.14 × 10^14^						4.93 × 10^5^	4.14 × 10^14^	1.24 × 10^15^
8	Wood (t)	4.40 × 10^4^			2.52 × 10^1^	1.11 × 10^6^						2.52 × 10^1^	1.11 × 10^6^	3.33 × 10^6^
9	Aluminum (kg)	1.60 × 10^10^			3.03 × 10^2^	4.85 × 10^12^						3.03 × 10^2^	4.85 × 10^12^	1.45 × 10^13^
10	Plastic (g)	3.80 × 10^10^			1.35 × 10^2^	5.12 × 10^12^						1.35 × 10^2^	5.12 × 10^12^	1.54 × 10^13^
11	Sand (t)	1.69 × 10^12^			9.57 × 10^2^	1.62 × 10^15^						9.57 × 10^2^	1.62 × 10^15^	4.85 × 10^15^
12	Diesel (J)	1.21 × 10^5^	9.61 × 10^6^	1.16 × 10^12^	1.43 × 10^9^	1.73 × 10^14^	0	0	0	1.85 × 10^7^	2.24 × 10^12^	1.45 × 10^9^	1.76 × 10^14^	5.28 × 10^14^
13	Gasoline (J)	6.60 × 10^4^	1.06 × 10^7^	7.02 × 10^11^	3.82 × 10^8^	2.52 × 10^13^	0	0	0	6.59 × 10^7^	4.35 × 10^12^	4.59 × 10^8^	3.03× 10^13^	9.09 × 10^13^
14	Solar irradiation (J)	1.00	0	0	3.78 × 10^12^	3.78 × 10^12^	2.17 × 10^13 a^	4.34 × 10^14^	4.34 × 10^14^	0	0	4.38 × 10^14^	4.38 × 10^14^	1.31 × 10^15^
15	Water (J)	6.60 × 10^5^	6.49 × 10^8^	4.28 × 10^14^	5.84 × 10^8^	3.85 × 10^14^	6.00 × 10^11^	1.20 × 10^13^	7.92 × 10^18^	6.86 × 10^8^	4.53 × 10^14^	1.20 × 10^13^	7.92× 10^18^	2.38 × 10^19^
16	Electricity (J)	8.00 × 10^4^	1.17 × 10^9^	9.36 × 10^13^	1.78 × 10^9^	1.42 × 10^14^	4.22 × 10^12^	8.44 × 10^13^	6.75 × 10^18^	4.03 × 10^9^	3.22 × 10^14^	8.44 × 10^13^	6.75× 10^18^	2.02 × 10^19^
17	Labor (h)	1.36 × 10^13^	3.82 × 10^2^	5.20 × 10^15^	4.97 × 10^5^	6.76 × 10^18^	2.88 × 10^5^	5.76 × 10^6^	7.83× 10^19^	8.52 × 10^2^	1.16× 10^16^	6.26 × 10^6^	8.51 × 10^19^	2.55 × 10^20^
Total			5.72 × 10^15^ (0.01%)		1.59× 10^19^ (14.63%)		9.30 × 10^19^ (85.35%)			1.24 × 10^16^ (0.01%)		5.34 × 10^14^	1.09 × 10^20^	3.27 × 10^20^

^a^ Annual solar irradiation for O&M phase in this paper were calculated by Andrić, Pina [64].

**Table 6:** CO_2_ emissions in the rebuilding strategy.

Item	Resources (Unit)	Emission Factor (kg/unit)	Demolition	Emission	Construction	Emission	Annual	Annual Emission	O&M Emission	End-of-Life	Emission
1	Steel (kg)	2.208			5.30 × 10^5^	1.17 × 10^6^					
2	Cement (kg)	0.894			3.65 × 10^6^	3.27 × 10^6^					
3	Concrete (kg)	0.242			5.93 × 10^6^	1.44 × 10^6^					
4	Brick (kg)	0.2			9.03 × 10^5^	1.81 × 10^5^					
5	Lime (kg)	1.2			6.05 × 10^4^	7.25 × 10^4^					
6	Gravel (kg)	0.002			3.30 × 10^6^	6.61 × 10^3^					
7	Glass (kg)	1.4			2.32 × 10^3^	3.25 × 10^3^					
8	Wood (kg)	0.2			4.06 × 10^4^	8.12 × 10^3^					
9	Aluminum (kg)	1.407			3.98 × 10^2^	5.60 × 10^2^					
10	Sand (kg)	0.0069			1.50 × 10^6^	1.04 × 10^4^					
11	Diesel (L)	2.73	6.98 × 10^−1^	1.91	5.59 × 10^3^	1.53 × 10^4^	0	0	0	7.31 × 10^−1^	1.99
12	Gasoline (L)	2.26	6.17× 10^−1^	1.39	1.35 × 10^3^	3.06 × 10^3^	0	0	0	3.51 × 10^−1^	7.92 × 10^−1^
13	Electricity (kWh)	0.9229	8.08 × 10^2^	7.46 × 10^2^	4.97 × 10^4^	4.59 × 10^4^	8.66 × 10^5^	7.99 × 10^5^	4.79 × 10^7^	1.37 × 10^3^	1.26 × 10^3^
Total				7.49 × 10^2^		6.22 × 10^6^			4.79 × 10^7^		1.26 × 10^3^

**Table 7:** CO_2_ emissions in the refurbishment strategy.

Item	Resources (Unit)	Emission Factor (kg/unit)	Demolition	Emission	Construction	Emission	Annual	Annual Emission	O&M Emission	End-of-Life	Emission
1	Steel (kg)	2.208			2.04 × 10^5^	4.50 × 10^5^					
2	Cement (kg)	0.894			1.43 × 10^6^	1.28 × 10^6^					
3	Concrete (kg)	0.242			2.19 × 10^6^	5.31 × 10^5^					
4	Brick (kg)	0.2			1.79 × 10^5^	3.59 × 10^4^					
5	Lime (kg)	1.2			5.42 × 10^5^	6.51 × 10^5^					
6	Gravel (kg)	0.002			1.49 × 10^6^	2.99 × 10^3^					
7	Glass (kg)	1.4			4.93 × 10^2^	6.90 × 10^2^					
8	Wood (kg)	0.2			2.52 × 10^4^	5.04 × 10^3^					
9	Aluminum (kg)	1.407			3.03 × 10^2^	4.26 × 10^2^					
10	Sand (kg)	0.0069			9.57 × 10^5^	6.61 × 10^3^					
11	Diesel (L)	2.73	3.42× 10^−1^	9.35× 10^−1^	5.08 × 10^1^	1.39 × 10^2^	0	0	0	6.59 × 10^−1^	1.80
12	Gasoline (L)	2.26	2.89× 10^−1^	6.53× 10^−1^	1.04 × 10^1^	2.45 × 10^1^	0	0	0	1.79	4.05
13	Electricity (kWh)	0.9229	3.25 × 10^2^	3.00 × 10^2^	4.94 × 10^2^	4.56 × 10^2^	1.17 × 10^6^	1.08 × 10^6^	2.16 × 10^7^	1.12 × 10^3^	1.03 × 10^3^
Total				3.02 × 10^2^		2.97 × 10^6^			2.16 × 10^7^		1.04 × 10^3^

**Table 8:** Total emergy and total CO_2_ emissions in the three strategies.

Emergy	Emergy (seJ)	CO_2_ Emission (kg)
Rebuilding strategy	4.07 × 10^20^	5.41 × 10^7^
Reference strategy	2.87 × 10^20^	9.16 × 10^7^
Refurbishment strategy	3.27 × 10^20^	7.38 × 10^7^

**Table 9:** Emergy-based indicators.

Emergy Indices	Rebuilding Strategy	Reference Strategy	Refurbishment Strategy
R	3.87 × 10^19^	5.93× 10^18^	1.47× 10^19^
N	4.20× 10^18^	0.00	1.90× 10^18^
F	3.64 × 10^20^	7.24× 10^19^	9.24× 10^19^
Y	4.07 × 10^20^	7.83× 10^19^	1.09 × 10^20^
EYR	1.12	1.08	1.18
ELR	9.51	12.21	6.43
ESI	0.12	0.09	0.18

## Data Availability

The data presented in this study are available on request from the corresponding author.

## Nomenclature

Acronyms			
BIM	Building information modelling	i	material i
CO_2_	Carbon dioxide	LCA	Life cycle assessment
Em	Emergy	LCC	Life cycle cost
EM-LCA	Combination of emergy and LCA method	N	Nonrenewable resources
ELR	Environmental loading ratio	O&M	Operation and maintenance phase
ESI	Emergy sustainability index	R	Renewable resources
EYR	Emergy yield ratio	RC	Reinforced concrete
F	Emergy feedback	Y	Emergy yield
GHG	Greenhouse gas		
Variables and parameters			
$c_{d}$	Calorific value of diesel fuel (J)	$N_{h}$	Number of working hours per day of one employee (h)
$c_{g}$	Calorific value of gasoline (J)	$N_{n}$	Number of employees
$E_{CO_{2}-eq}$	Emission factor of the different building materials i (kg/unit)	S	construction site surface (footprint) (m^2^)
$E_{d}$	Solar emergy of the diesel fuel consumed (seJ)	$t_{c}$	Construction time (year)
$E_{e}$	Solar emergy of the electricity required in the building construction, O&M and end-of-life phases (seJ)	$T_{d}$	Transformity of diesel fuel (seJ/unit)
$E_{g}$	Solar emergy of gasoline (seJ)	$T_{e}$	Transformity of electricity (seJ/unit)
$E_{l}$	Solar emergy equivalent of human labor (seJ)	$T_{g}$	Transformity of gasoline (seJ/unit)
$E_{m}$	Solar emergy of the building materials (seJ)	$T_{l}$	Transformity of labor (seJ/unit)
$E_{s,c}$	Solar emergy of the solar irradiation in the construction phase (seJ)	$T_{mi}$	Transformity of material i (seJ/unit)
$E_{w,c}$	Solar emergy of the water consumed in the building demolition, construction and end-of-life phases (seJ)	$T_{s}$	Transformity of solar energy (seJ/unit)
$E_{w,o}$	Solar emergy of the water consumed in the building O&M phase (seJ)	$T_{w}$	Transformity of water (seJ/unit)
G	Gibbs free energy of water (J/g)	$t_{o}$	Total number of working days per year (day)
I	Annual amount of solar radiation ($J/m^{2}$)	$t_{t}$	Working days required in the life cycle process (day)
$G_{CO_{2}}$	Amount of CO_2_ emissions (kg)	$V_{a}$	Required water volume of one person per day (m^3^)
$M_{d}$	Quantity of diesel fuel consumed in the building life cycle (L)	V	Required water volume (m^3^)
$M_{g}$	Quantity of gasoline consumed in the building life cycle (L)	W	Quantity of electricity (kWh)
$M_{i}$	Quantity of material i	ρ	Water density (kg/m^3^)
